# A novel approach for fibrous dysplasia assessment using combined planar and quantitative SPECT/CT analysis of Tc-99m-diphosphonate bone scan in correlation with biological bone turnover markers of disease activity

**DOI:** 10.3389/fmed.2022.1050854

**Published:** 2022-11-25

**Authors:** Mario Jreige, Nicolas Hall, Fabio Becce, Bérengère Aubry-Rozier, Elena Gonzalez Rodriguez, Niklaus Schaefer, John O. Prior, Marie Nicod Lalonde

**Affiliations:** ^1^Department of Nuclear Medicine and Molecular Imaging, Lausanne University Hospital and University of Lausanne, Lausanne, Switzerland; ^2^Interdisciplinary Centre for Bone Diseases, Service of Rheumatology, Lausanne University Hospital and University of Lausanne, Lausanne, Switzerland; ^3^Department of Diagnostic and Interventional Radiology, Lausanne University Hospital and University of Lausanne, Lausanne, Switzerland

**Keywords:** fibrous dysplasia, SPECT/CT, scintigraphy, bone scan, quantitative imaging, bone turnover markers

## Abstract

**Purpose:**

To investigate the emerging role of Tc-99m-labeled diphosphonate (Tc-99m-DPD) uptake quantification by SPECT/CT in fibrous dysplasia (FD) bone lesions and its correlation with biological bone turnover markers (BTMs) of disease activity.

**Materials and methods:**

Seven patients (49 ± 16 years) with a confirmed diagnosis of FD were included in this retrospective study. Bone scans with Tc-99m-DPD and quantitative SPECT/CT (xSPECT/CT) were performed. SUV_max_ (maximum standard unit value) and SUV_mean_ (mean standard unit value) were measured in all FD bone lesions. The skeletal burden score (SBS) was assessed on planar scintigraphy and multiplied by mean SUV_*max*_ and SUV_mean_ to generate two new parameters, SBS_SUV_max_ and SBS_SUV_mean_, respectively. Planar and xSPECT/CT quantitative measures were correlated with biological BTMs of disease activity, including fibroblast growth factor 23 (FGF-23), alkaline phosphatase (ALP), procollagen 1 intact N-terminal propeptide (P1NP) and C-terminal telopeptide (CTX), as well as scoliosis angle measured on radiographs. Statistical significance was evaluated with Spearman’s correlations.

**Results:**

A total of 76 FD bone lesions were analyzed, showing an average SUV_max_ and SUV_mean_ (g/mL) of 13 ± 7.3 and 8 ± 4.5, respectively. SBS, SBS_SUV_max_ and SBS_SUV_mean_ values were 30.8 ± 25.6, 358 ± 267 and 220.1 ± 164.5, respectively. Mean measured values of FGF-23 (pg/mL), ALP (U/L), P1NP (μg/L) and CTX (pg/mL) were 98.4 (22–175), 283.5 (46–735), 283.1 (31–1,161) and 494 (360–609), respectively. Mean scoliosis angle was 15.7 (7–22) degrees. We found a very strong positive correlation between planar-derived SBS and CTX (*r* = 0.96, *p* = 0.010), but no significant correlation between SUV_max_ or SUV_mean_ and biological BTMs. SBS_SUV_max_ showed a strong to very strong positive correlation with CTX (ρ = 0.99, *p* = 0.002), FGF-23 (ρ = 0.91, *p* = 0.010), ALP (ρ = 0.82, *p* = 0.020), and P1NP (ρ = 0.78, *p* = 0.039), respectively.

**Conclusion:**

This study showed that biological BTMs are significantly correlated with diphosphonate uptake on bone scan, quantified by a new parameter combining information from both planar and quantitative SPECT/CT. Further analysis of bone scan quantitative SPECT/CT data in a larger patient population might help better characterize the skeletal disease burden in FD, and guide treatment and follow-up.

## Introduction

Fibrous dysplasia (FD) is a benign, non-hereditary congenital bone disorder caused by impaired osteogenesis secondary to an activating somatic mutation in the GNAS gene, leading to mutations in the alpha subunit of the Gs protein (1, 2). FD is characterized by intramedullary fibro-osseous proliferative lesions and may present in a monostotic (single bone) or polyostotic (multiple bones) form, or as a feature of two rare syndromes, namely the McCune-Albright syndrome or the Mazabraud syndrome (1, 3). Symptoms of FD include bone pain, fractures, bone deformities and neurological deficits (4). Bone turnover markers (BTMs), namely alkaline phosphatase (ALP), procollagen 1 intact N-terminal propeptide (P1NP) and C-terminal telopeptide (CTX), have been used as markers of disease activity, but serum levels may be influenced by age, comorbidities and treatments, including bisphosphonates and denosumab (2, 5, 6). FGF-23 is a phosphate-regulating hormone overproduced in FD lesions which levels correlate with the disease burden and can also be used as a biomarker of disease activity (7). The skeletal burden score (SBS) derived from planar ^99^Tc-methylene diphosphonate (^99^Tc-MDP) bone scan evaluation has been validated as a reliable instrument for measuring the global skeletal burden of FD, based on estimation of the percentage of affected skeleton area on whole-body images. However, SBS derived from ^99^Tc-MDP is limited as it is a semiquantitative method based on two-dimensional imaging (8). To overcome these limitations, Van der Bruggen et al. proposed to quantify the skeletal burden in FD using sodium fluoride PET/CT (9). Their study showed a strong correlation of Na^18^F-PET/CT FD burden measurements with biological BTMs and suggested a possible role of this technique in treatment follow-up. The major limitation of Na^18^F-PET/CT is its low availability and high cost, as opposed to bone scintigraphy. Recently, technological advances enabled to quantify ^99m^Tc-DPD uptake in single-photon emission computed tomography (SPECT) coupled with computed tomography (CT) (xSPECT/CT, Symbia Intevo, Siemens Healthineers, Erlangen, Germany). The xSPECT showed an accurate activity recovery within 10% of the expected value for objects > 10 mL, which is similar to PET/CT (10).

The aim of our study was to investigate the correlation between Tc-99m-DPD xSPECT/CT uptake quantification of FD bone lesions and biological BTMs and scoliosis, as a complication of FD.

## Materials and methods

### Patient selection

Between 2016 and 2021, 17 patients with a confirmed diagnosis of FD were treated in our Interdisciplinary Centre for Bone Diseases at Lausanne University Hospital. Of these, 2 patients were excluded from this retrospective analysis because they did not have bone scintigraphy and 8 because they did not undergo quantitative SPECT/CT. Hence, our final study population consisted of 7 patients: 2 males and 5 females, with a median age of 59 years (range, 26–72 years), with confirmed diagnosis of FD based on histopathology or as part of a clinical syndrome (Mazabraud or McCune-Albright syndromes), available BTMs values and Tc-99m-DPD xSPECT/CT images. We retrospectively collected clinical, biological and radiological data from the patients’ hospital medical records. The local Ethics Research Committee of the State of Vaud approved the research protocol (CER-VD #2018-01513). All patients participating in this study had signed an institutional general consent for retrospective use of their data in clinical research.

### Biological bone turnover markers measurements

Dosage of biological markers were performed on early morning fasting blood samples. Results of the most recent BTMs dosage in respect to bone imaging were used. ALP, P1NP and CTX were measured in the central clinical routine laboratory of the Lausanne University Hospital; intact FGF-23 was measured by ELISA (Kainos, Tokyo, Japan) at the Inselspital (Bern, Switzerland). For P1NP and CTX premenopausal women normal ranges are used as reference (P1NP: < 58.6 μg/L; CTX: < 573 ng/L). Normal values are 36–120 UI/L for ALP, and 10–50 pg/mL for FGF-23.

### Tc-99m-labeled diphosphonate single-photon emission computed tomography/computed tomography acquisition and analysis

All patients underwent whole-body planar imaging with low-energy high-resolution collimators, and a scanning speed of 12 cm/min, followed by quantitative SPECT/CT (Symbia Intevo, Siemens Healthineers, Erlangen, Germany) on regions with high uptake on planar scintigraphy. The xSPECT was acquired in average at 3 h and 22 min ± 31 min after intravenous injection of 10 MBq/kg of 99mTc-DPD with a mean patient dose of 798 ± 58 MBq. Images were acquired with 3 degrees rotation/step and 12 s/projection with a 256 × 256 matrix. Reduced-dose CT was acquired using 120 kV and 25 reference mAs modulation (Siemens Care Dose, Symbia Intevo, Erlangen, Germany). Images were reconstructed to generate xSPECT data allowing SUVbw quantification on post-processed images and measurement of SUV_max_ and SUV_mean_ (g/mL) using xSPECT reconstruction algorithm.

**FIGURE 1:**
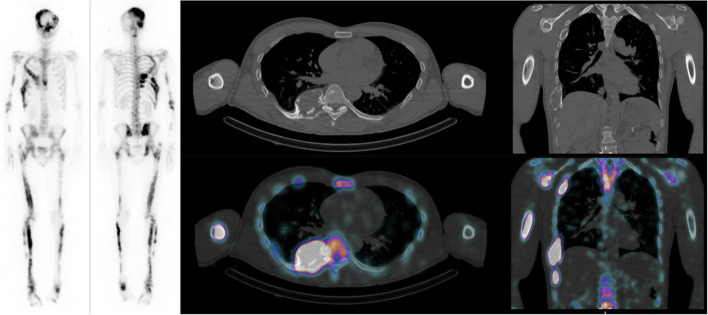
Forty-year-old male patient with Mazabraud syndrome: Mean SUV_max_ and SUV_mean_ of 11 and 6.7 g/mL, respectively. SBS: 46.3, FGF-23: 175 (10–50 pg/mL), CTX: 531 pg/mL, P1NP: 383 (15–78 μg/L), ALP: 277 (36–108) U/L, scoliosis angle: 11 degrees.

For each patient, the SUV_max_ and SUV_mean_ of all FD bone lesions visible on xSPECT and CT were measured with a 42% thresholding and classified according to their location in the axial or appendicular skeleton (Figure 1). All FD bone lesions were visually assessed based on pathological high uptake, excluding uptake due to degenerative changes. The SBS was assessed on planar scintigraphy for all patients in consensus by two nuclear medicine specialists (MJ and MNL) as described by Collins et al., and SBS_SUV_max_ and SBS_SUV_mean_ were generated by multiplying SBS by mean SUV_max_ and SUV_mean_ of all lesions for each patient, respectively (8).

Scoliosis Cobb angles were measured using the Carestream Vue PACS’s orthopedics workflow tool by a musculoskeletal radiologist.

### Statistical analysis

Continuous variables are reported as mean ± standard deviation (SD) and range.

Categorical data were analyzed using Fisher’s exact test or chi-squared test, as appropriate. For comparison of two groups, the Student’s *t*-test was used when assumptions for parametric tests were met; otherwise, the Mann-Whitney *U*-test was used.

Planar SBS and all xSPECT/CT quantitative measures were correlated with biological BTMs of disease activity, including FGF-23, ALP, P1NP and CTX, as well as with scoliosis angle using Spearman’s correlation. Statistical analysis was performed using STATA (version 14.0; STATA Corp., College Station, Texas, USA). *P*-values less than 0.05 were considered as statistically significant.

## Results

### Study population

Seven patients with confirmed FD, quantitative SPECT/CT and BTMs dosage were retrospectively identified and included in this study. They had been referred for evaluation of FD confirmed on histopathological analysis (*n* = 6) and/or within the spectrum of a known syndrome (*n* = 3, 1 McCune-Albright syndrome and 2 Mazabraud syndromes). One patient with McCune-Albright syndrome did not have histopathological confirmation (Table 1). FD lesions were assessed on Tc-99m-DPD xSPECT/CT in all 7 patients. All seven included patients had a polyostotic form of FD. Four out of seven (57%) patients had previously been treated with bisphosphonates. A total number of 76 lesions for all patients were measured on xSPECT/CT with a mean number of lesions per patient of 10.9 ± 9.6. No difference was observed in terms of distribution of the 76 lesions between the axial (*n* = 50) and appendicular (*n* = 26) skeleton (*p* = 0.273).

**TABLE 1:** Patient characteristics.

Patient number	Age (years)	Sex	Syndrome	Lesion location	Number of analyzed lesions	Scoliosis angle (degree)	Previous fractures	Treatment
1	48	Female	McCune-Albright	Axial and appendicular	10	22	Right 3rd, 6th, and 7th ribs	Bisphosphonate followed by denosumab
2	34	Female	–	Appendicular	1	7	–	–
3	26	Female	–	Axial and appendicular	4	11	–	Bisphosphonate
4	39	Male	Mazabraud	Axial and appendicular	13	11	–	–
5	62	Female	Mazabraud	Appendicular	5	21	–	Bisphosphonate
6	72	Male	–	Axial and appendicular	30	19	Right femoral diaphysis	–
7	55	Female	–	Axial and appendicular	13	19	–	Bisphosphonates

### Biological bone turnover markers

Mean measured values of biological BTMs were: FGF-23, 98.4 ± 56.3 pg/mL (22–175); ALP, 283.5 ± 243.6 U/L (46–735); P1NP, 283.1 ± 406.7μg/L (31–1,161); and CTX, 494 ± 90.5 pg/mL (360–609). Scoliosis was reported in all patients with a mean angle of 15.7 ± 5.9 (7–22) degrees.

### Quantitative Tc-99m-labeled diphosphonate single-photon emission computed tomography/computed tomography

A total number of 76 FD bone lesions were analyzed, showing a mean SUV_max_ and SUVmean of 13 ± 7.3 and 8 ± 4.5 g/mL, respectively. Mean SBS score was 30.8 ± 25.6. Mean SBS_SUV_max_ and SBS_SUV_mean_ were 358 ± 267 and 220 ± 165 g/mL, respectively. We found significantly higher values of SUV_max_ and SUV_mean_ in axial skeleton lesions (14.5 ± 1.1 and 8.9 ± 0.6 g/mL, respectively) compared to appendicular skeleton lesions (10 ± 1.2 and 6.2 ± 0.8 g/mL, respectively) (*p* = 0.010 and *p* = 0.013, respectively).

### Correlations between bone turnover markers and imaging quantification

**FIGURE 2:**
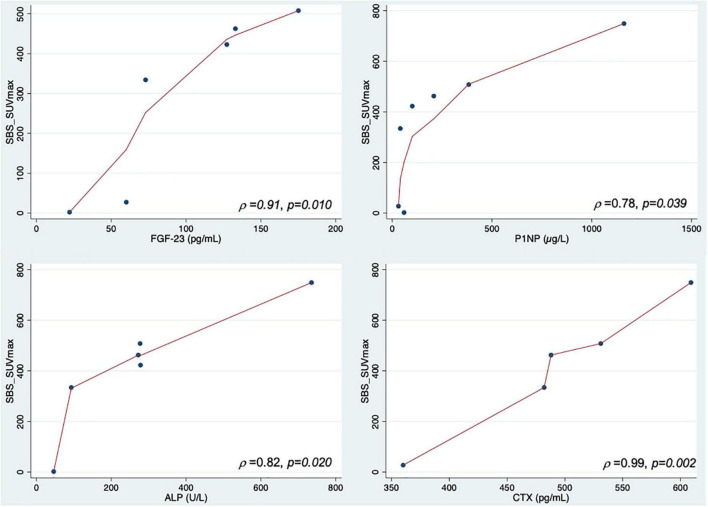
Correlations between SBS_SUV_max_ and FGF-23, ALP, P1NP and CTX.

Spearman correlations results are shown in Table 2. We found a very strong positive correlation between planar-derived SBS and CTX (ρ = 0.96, *p* = 0.01), a statistical trend for a positive correlation with FGF-23 (ρ = 0.81, *p* = 0.053) and ALP (ρ = 0.79, *p* = 0.060) but no correlation with P1NP (ρ = 0.69, *p* = 0.089). No significant correlation was found between SUV_max_ or SUV_mean_ and biological BTMs. Combining both SBS and SUV increased the strength of the correlations: SBS_SUV_max_ and SBS_SUV_mean_ showed a strong to very strong positive correlation with CTX (ρ = 0.99, *p* = 0.002 and ρ = 0.99, *p* = 0.001), FGF-23 (ρ = 0.91, *p* = 0.010 and ρ = 0.91, *p* = 0.013), ALP (ρ = 0.82, *p* = 0.020 and ρ = 0.88, *p* = 0.019), and P1NP (ρ = 0.78, *p* = 0.039 and ρ = 0.78, *p* = 0.028), as shown in Figure 2. There was no correlation between bone scan quantitative measures and scoliosis angle. No significant correlation was found between SUV_max_ or SUV_mean_ of axial skeleton FD lesions with scoliosis angle (ρ = 0.33, *p* = 0.472 and ρ = 0.36, *p* = 0.432, respectively).

**TABLE 2:** Correlations between bone scintigraphy quantitative measures and biological bone turnover markers of disease activity and scoliosis angle.

Spearman correlations (ρ, CI, *p*-value)	SUV_max_	SUV_mean_	SBS	SBS_SUV_max_	SBS_SUV_mean_
FGF-23	(0.28, –0.69–0.89, 0.585)	(0.29, –0.68–0.89, 0.581)	(0.81, –0.02–0.98, 0.053)	**(0.91, 0.40–0.99, 0.010)**	**(0.91, 0.36–0.99, 0.013)**
ALP	(0.01, –0.81–0.81, 0.994)	(0.01, –0.81–0.81, 0.997)	(0.79, –0.05–0.98, 0.060)	**(0.82, 0.25–0.99, 0.020)**	**(0.88, 0.26–0.99, 0.019)**
P1NP	(0.04, –0.73–0.77, 0.928)	(0.06, –0.73–0.77, 0.901)	(0.69, –0.14–0.95, 0.089)	**(0.78, 0.06–0.97, 0.039)**	**(0.78, 0.07–0.97, 0.028)**
CTX	(0.13, –0.85–0.91, 0.837)	(0.18, –0.84–0.92, 0.772)	**(0.96, 0.50– 0.99, 0.010)**	**(0.99, 0.83–0.99, 0.002)**	**(0.99, 0.85–0.99, 0.001)**
Scoliosis angle	(0.33, –0.56–0.87, 0.472)	(0.36, –0.52–0.88, 0.432)	(0.58, –0.31–0.93, 0.168)	(0.62, –0.25–0.94, 0.137)	(0.63, –0.24–0.94, 0.133)

CI, Confidence interval; SUV, Standard uptake value; SUVmax, maxium Standard Uptake Value; SUVmean, mean Standard Uptake Value; SBS, Skeletal Burden Score; SBS_SUVmax, SBS score multiplied by SUVmax; SBS_SUVmean, SBS score multiplied by SUVmean; FGF-23, Fibroblast growth factor 23; ALP, Alkaline phosphatase; P1NP, Procollagen 1 intact N-terminal propeptide; CTX, C-terminal telopeptide.

## Discussion

In this study, we propose a novel approach to quantify the FD disease burden on Tc-99m-DPD bone scan using a combination of planar and absolute SPECT/CT quantification by multiplying the well-known SBS score by the average SUV_max_ and SUV_mean_ values of the patient’s FD lesions, leading to a disease-extension-corrected SUV value, which we termed SBS_SUV_max_ and SBS_SUV_mean_, respectively. We showed that SBS_SUV_max_ and SBS_SUV_mean_ improved the correlation of bone imaging with disease activity as compared to SBS and SUV values alone: correlations between the results of this combined quantitative bone scan approach and biological activity as assessed by BTMs were strong to very strong and highly statistically significant.

To differentiate monostotic from polyostotic forms of FD and to evaluate the disease extent, patients with FD routinely undergo a Tc-99m-diphosphonate bone scan as recommended by current guidelines (11). Collins et al. have developed and validated the SBS, which is derived from a weighted score based on the estimation of the amount of FD lesions in anatomical segments on bone scintigraphy (8). The SBS correlated positively with BTMs, such as ALP, bone-specific ALP, osteocalcin, pyridinium cross-links, deoxypyridinoline cross-links, and N-telopeptide (8). However, this score has its limitations as it remains semiquantitative and is unable to quantify bone turnover activity within individual FD lesions, which may be of clinical relevance. ^18^F-NaF-PET/CT overcomes this limitation by allowing single lesion bone turnover quantification (12). Van der Bruggen et al. demonstrated that 18F-NaF-PET/CT SUV thresholds could discriminate healthy bone from FD lesions (9). In addition, their study showed a strong relationship between serum markers of bone formation and measurements of FD burden by ^18^F-NaF-PET/CT. Whereas SBS is not influenced by medical treatment, total lesion fluorination was higher in patients at baseline than in patients treated with biphosphonates, implying a potential role for Na^18^F-PET/CT in treatment response assessment (9). Papadakis et al. also investigated the role of ^18^F-NaF-PET/CT in FD skeletal burden assessment, and showed that BTMs, including ALP, N-telopeptides, and osteocalcin, were strongly correlated with total volume of all ^18^F-NaF-positive FD lesions. These authors also found a very strong correlation between SBS derived from ^18^F-NaF PET/CT and ^99m^Tc-MDP scintigraphy (13). The drawback of ^18^F-NaF PET/CT is that it is not reimbursed in many countries and/or not readily available, constituting an obstacle to its use in routine clinical practice.

Recently, absolute quantification of bone scintigraphy has become available and has been the subject of several studies. For example, Yamane et al. showed in a pediatric population that SUV was higher at the epiphyseal plates of children under 15 years of age compared to older subjects, consistent with higher osteoblastic activity (14). Another study in an adult population showed a significant difference in quantitative ^99m^Tc-DPD uptake on bone xSPECT/CT between prostate cancer bone metastases and spinal and pelvic osteoarthritic changes, with higher SUV_max_ and SUV_mean_ in metastases (15). Therefore, the quantification of radiotracer uptake on bone scan might help differentiating between benign and malignant pathologies or other rheumatological disorders with high bone turnover, which needs to be confirmed by prospective studies.

To the best of our knowledge, our study is the first to report the absolute quantification of ^99m^Tc-DPD uptake on xSPECT/CT in FD. We introduced new quantitative parameters, SBS_SUV_max_ and SBS_SUV_mean_, to evaluate the disease burden. The main motivation for this quantitative model was to optimize the information extracted from bone scintigraphy combining both the overall disease activity assessed by SBS on a whole-body basis and the degree of tracer uptake assessed by mean SUV measurements of lesions in the xSPECT/CT field of view, as xSPECT/CT was not acquired on the whole body.

In our study, SBS alone correlated only with CTX, with a statistical trend toward a positive association with FGF-23 and ALP, while SUV_max_ and SUV_mean_ did not correlate with any BTMs. In contrast, SBS_SUV_max_ and SBS_SUV_mean_ correlated with all four BTMs. Multiplying mean SUV_max_ and SUV_mean_ of all lesions on xSPECT/CT by SBS could allow a more accurate characterization of bone turnover and disease activity in FD. The advantage of this quantification method is that bone scan is widely available and SPECT/CT quantification methods are increasingly implemented with new gamma cameras.

Finally, ^18^F-FDG PET/CT has also been studied in FD and has shown a significant role in detecting malignant transformation of FD and optimizing patient management strategies in a complementary manner to ^99m^Tc-MDP SPECT/CT (16). Therefore, quantification of bone scintigraphy may increase the accuracy of SPECT/CT in predicting disease activity, allowing this modality to remain competitive in the era of novel multimodal imaging, with particular potential as a biomarker for assessing disease activity and for monitoring treatment response.

This is a preliminary retrospective study, and thus its main limitation is the small number of patients included. However, the relatively large number of FD lesions analyzed increased the accuracy of the bone scan scores calculated for each patient. Further analyses of larger patient populations are needed to confirm the observed correlation between bone scintigraphy quantification and biological BTMs. In this study, SUV thresholding was used with a standard level of 42%, limiting reproducibility issues. However, an optimized approach to lesion delineation should be evaluated in larger future studies and compared with a standard fixed-level thresholding approach. In our study, the values of the scintigraphic quantitative measures did not correlate with the scoliosis angle. Interpretation of this observation remains limited as the range of scoliosis angles measured in our population was low.

## Conclusion

This preliminary study showed a significant correlation between biological BTMs of disease activity and diphosphonate uptake on bone scan, quantified by a new parameter combining information from both planar and quantitative SPECT/CT. This approach could become the routine technique for clinical assessment of skeletal burden in FD due to its widespread availability. Further analysis of bone scan quantitative SPECT/CT data might help better characterize the skeletal disease burden in FD, and its role in guiding treatment and follow-up.

## Data availability statement

The raw data supporting the conclusions of this article will be made available by the authors, without undue reservation.

## Ethics statement

The studies involving human participants were reviewed and approved by the Ethics Research Committee of the State of Vaud. Written informed consent for participation was not required for this study in accordance with the national legislation and the institutional requirements.

## Author contributions

MJ and MN designed the work, performed and interpreted the data analysis, and drafted and revised the work. NH participated to data collection. FB performed the radiologic measurements and revised the work. BA-R, EG, NS, and JP critically revised the work. All authors contributed to the article and approved the submitted version.
