# Erratum: A novel approach for fibrous dysplasia assessment using combined planar and quantitative SPECT/CT analysis of Tc-99m-diphosphonate bone scan in correlation with biological bone turnover markers of disease activity

**DOI:** 10.3389/fmed.2023.1171916

**Published:** 2023-03-07

**Authors:** Frontiers Production Office

**Affiliations:** Frontiers Media SA, Lausanne, Switzerland

**Keywords:** fibrous dysplasia, SPECT/CT, scintigraphy, bone scan, quantitative imaging, bone turnover markers

An omission to the funding section of the original article was made in error. The following sentence has been added: “Open access funding was provided by the University of Lausanne.”

The original article has been updated.

